# Supplementary material for: Declines over the last two decades of five intertidal invertebrate species in the western North Atlantic
Source: Commun Biol. 2020 Oct 20;3:591. doi: 10.1038/s42003-020-01326-0 (PMC7576203; doi:10.1038/s42003-020-01326-0)
Supplement: Supplementary file 1 — Supplementary Information [file 42003_2020_1326_MOESM1_ESM.pdf]

Supplementary Fig. 1: Monthly mean temperatures (°C) from 2002 to 2018.

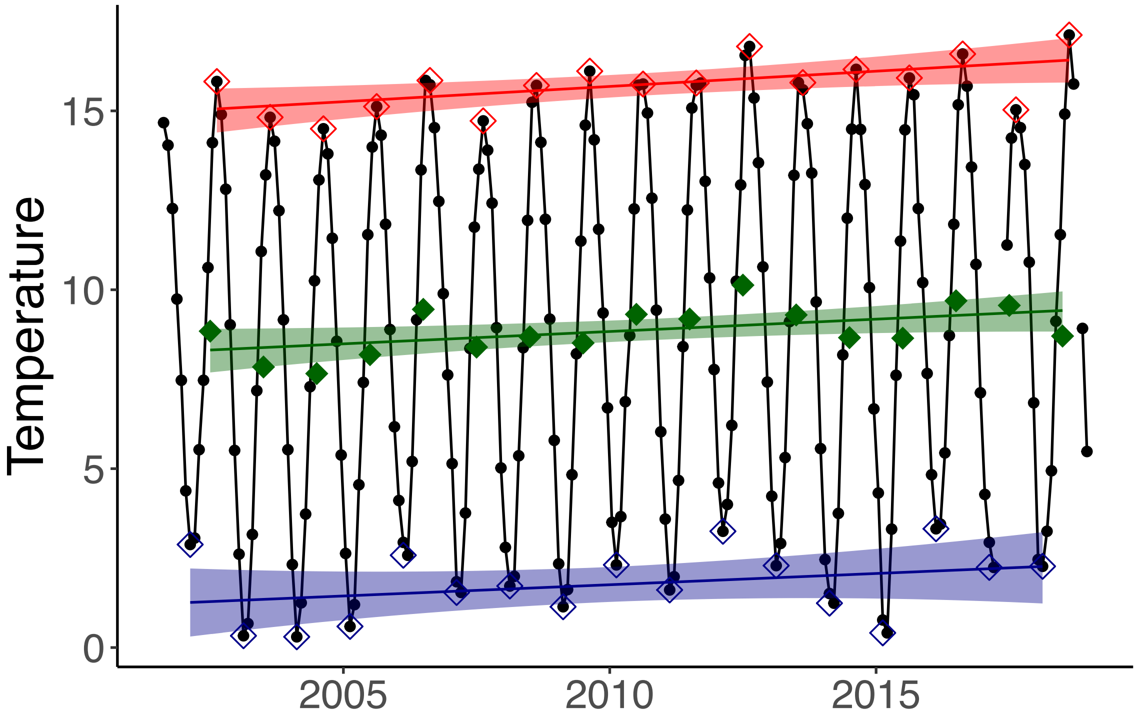

Sinusoidal curve is plotted through the monthly means (black dots). Data for monthly means can be found in Supplementary Data 1. Lines are Bayesian estimates of slopes and intercepts, and shaded regions are 95% credible intervals (see Supplementary Table 6 for details). Red regression line and shading are for early maximum temperature, blue is yearly minimum and green is yearly average.

Supplementary Table 1. Bayesian estimates of fixed effects from Poisson regressions of abundances and recruitment over time and shown in Figure 1. Models done using number per m^2 for LL, LO, TT and NL; square root of number per 36 cm^2 for SB; number per sampling unit (40 cm^2) for ME. Codes are: LL=*L. littorea*, LO=*L. obtusata*, TT=*T. testudinalis*, NL=*N. lapillus*, SB=*S. balanoides,* ME=*M. edulis.* Column labeled pMCMC gives the proportion of runs for which the estimate of the posterior mean was ≤ 0.

Species	Term	posterior mean	lower 95% credible limit	upper 95% credible limit	effective sample size	pMCMC
LL	Intercept	69.140	47.543	89.589	2,000	0.0005
	Year	-0.032	-0.042	-0.022	2,000	0.0005
LO	Intercept	-17.303	-54.074	19.280	2,000	0.3430
	Year	0.010	-0.008	0.029	2,000	0.2750
TT	Intercept	125.298	60.452	205.229	2,151	0.0005
	Year	-0.063	-0.098	-0.026	2,149	0.0005
NL	Intercept	76.896	26.902	131.423	2,000	0.0070
	Year	-0.038	-0.065	-0.013	2,000	0.0070
SB	Intercept	50.800	6.420	103.455	2,000	0.0420
	Year	-0.025	-0.051	-0.003	2,000	0.0440
ME	Intercept	319.058	271.294	371.278	2,000	0.0005
	Year	-0.157	-0.183	-0.134	2,000	0.0005

Supplementary Table 2. Percent change per year and half-life in years for gastropod abundances and recruitment by mussels and barnacles based on data in Table 1. See Table 1 for codes for species. Note that Poisson estimates for barnacle recruits in Table 1 are based on square root transformed data, and so values in this table were back-transformed.

	Percent change per year			Half-life in years		
Species	posterior mean	lower 95% credible limit	upper 95% credible limit	posterior mean	lower 95% credible limit	upper 95% credible limit
LL	-3.2%	-4.2%	-2.2%	21	16	32
LO	1.0%	-0.8%	2.9%	-70	91	-24
TT	-6.3%	-9.8%	-2.6%	11	7	27
NL	-3.8%	-6.5%	-1.3%	18	11	53
SB	-5.0%	-10.2%	-0.5%	14	7	136
ME	-15.7%	-18.3%	-13.4%	4	4	5

Supplementary Table 3. Best supported models of the effects on environmental factors on abundance and recruitment. Estimates are based on pooled estimates from 10 runs of imputed values and so differ from estimates in Supplementary Tables 4 and 5, which dropped missing values. See Table 1 for species codes. Results are pooled estimates based on imputed values for environmental data. Results of LL, LO, NL and TT are numbers per m^2; barnacle (SB) data are square root transform of number per 36 cm^2; mussel (ME) data are log_{10} transform of counts per 40 cm^2. T=temperature, and Ω = saturation ratio.

Species	Parameter	Estimate	SE	z value	P
LL	Intercept	534.00	91.10	5.86	>0.0001
	One-year lag in T	-49.30	10.30	-4.81	>0.0001
LO	Intercept	2427.40	3300.00	0.74	0.4620
	One-year lag in Ω	-186.00	62.60	-2.97	0.0029
	One-year lag in pH	-254.60	408.90	-0.62	0.5335
	Intercept	371.60	117.80	3.15	0.0016
	One-year lag in Ω	-182.10	61.90	-2.94	0.0032
TT	Intercept	17.54	5.37	3.27	0.0011
	One-year lag in Ω	-8.43	2.82	-2.99	0.0028
NL	Intercept	74.40	26.20	2.84	0.0046
	One-year lag in Ω	-36.30	13.80	-2.64	0.0083
ME	Intercept	8.08	1.56	5.20	>0.0001
	T in August	-0.38	0.15	-2.44	0.0150
	T in July	-0.05	0.13	-0.39	0.6950
	Intercept	8.19	1.48	5.54	>0.0001
	T in August	-0.43	0.09	-4.54	>0.0001
SB	Intercept	-89.25	777.53	-0.11	0.9100
	T in February	0.53	1.75	0.30	0.7600
	T in March	-0.86	1.71	-0.50	0.6100
	Current year pH	11.81	96.66	0.12	0.9000
	Intercept	5.47	1.43	3.83	0.0001
	T in February	-0.33	0.66	-0.51	0.6111
	Intercept	5.86	1.51	3.87	0.0001
	T in March	-0.47	0.65	-0.72	0.4739

Supplementary Table 4. Parameter estimates for the best-supported models of regressions of snail abundances versus environmental data. Based on data in Supplementary Data 1. Column abbreviations are: Spp = species ID, Int = intercept, $\boldsymbol{\Omega}$ = saturation ratio, pH = pH, T = water temperature, LL = log-likelihood ratio, AICc = corrected Akaike information criterion, delta = Akaike delta, Wt = Akaike weight. Species IDs are: LL=*L. littorea*, LO=*L. obtusata*, NL=*N. lapillus*, TT=*T. testudinalis*. Subscripts denote: 0 = current year, 1 = previous year.

**Spp**	**Int**	$\boldsymbol{\Omega_0}$	$\boldsymbol{\Omega_1}$	$\mathbf{pH_0}$	$\mathbf{pH_1}$	$\mathbf{T_0}$	$\mathbf{T_1}$	**LL**	**AICc**	**delta**	**Wt**
LL	594.4						-54.8	-45.03	100.1	0.00	0.741
	-18840.0			2406.0			-45.6	-43.83	103.7	3.60	0.123
	-14860.0				1913.0		-47.6	-44.38	104.8	4.69	0.071
	-37410.0	-482.5			4779.0			-44.48	104.9	4.88	0.065
LO	-19180.0		-224.4		2442.0			-34.98	85.9	0.00	0.568
	238.5						-23.2	-39.44	88.9	2.93	0.131
	556.2	-275.3						-39.54	89.1	3.13	0.119
	720.4	-200.8	-161.9					-36.75	89.5	3.54	0.097
	487.4	-170.9					-14.7	-36.87	89.7	3.79	0.086
NL	7.1							-29.22	64.2	0.00	0.452
	72.8		-34.8					-28.12	66.2	2.08	0.160
	-2887.0			359.9				-28.36	66.7	2.56	0.125
	24.2						-1.9	-28.76	67.5	3.36	0.084
	36.9	-15.7						-29.01	68.0	3.87	0.065
	-1159.0				145.0			-29.10	68.2	4.05	0.060
	3.8					0.4		-29.21	68.4	4.26	0.054
TT	1.4							-10.76	27.2	0.00	0.272
	14.8		-7.1					-8.77	27.5	0.29	0.235
	13.8	-6.6						-9.08	28.2	0.92	0.171
	5.9						-0.5	-9.34	28.7	1.44	0.132
	5.4					-0.5		-9.80	29.6	2.36	0.084
	-260.0			32.5				-10.50	31.0	3.75	0.042
	-247.8				31.0			-10.54	31.1	3.84	0.040
	19.0	-4.2	-5.1					-8.03	32.1	4.82	0.024

Supplementary Table 5. Parameters for best-supported models of barnacle and mussel recruitment versus environmental data for delta less than five. Based on data in Supplementary Data 1. Column abbreviations are: Spp = species ID, Int = intercept, $\boldsymbol{\Omega}$ = saturation ratio, pH = pH, logL = log-likelihood ratio, AICc = corrected Akaike information criterion, delta = Akaike delta, Wt = Akaike weight. Species IDs are: SB = *S. balanoides*, ME = *M. edulis*.

Spp	Int	Feb	Mar	Apr	May	$\boldsymbol{\Omega}$	pH	logL	AICc	delta	Wt
SB	5.39							-27.48	60.50	0.00	0.485
	-1137.00						142.10	-26.91	63.20	2.79	0.120
	4.19	0.69						-27.17	63.80	3.32	0.092
	4.11		0.63					-27.24	63.90	3.46	0.086
	3.23			0.47				-27.36	64.10	3.70	0.076
	-2.85					4.33		-27.44	64.30	3.85	0.071
	3.52				0.23			-27.45	64.30	3.88	0.070

	Int	May	Jun	Jul	Aug	$\boldsymbol{\Omega}$	pH	logL	AICc	delta	Wt
ME	6.93				-0.35			1.00	8.00	0.00	0.262
	4.24			-0.19				0.24	9.50	1.52	0.123
	3.50	-0.24						0.23	9.50	1.53	0.122
	4.26		-0.23					-0.02	10.00	2.04	0.094
	3.99				-0.61	3.67		2.81	10.40	2.39	0.079
	-289.20						36.15	-0.32	10.60	2.64	0.070
	-169.30				-0.27		21.75	2.44	11.10	3.12	0.055
	-206.70			-0.15			26.15	2.36	11.30	3.28	0.051
	1.48							-2.86	11.40	3.43	0.047
	-198.20	-0.18					25.02	2.04	11.90	3.91	0.037
	-212.00				-0.56	4.36	26.60	6.49	12.00	4.03	0.035
	-198.10		-0.17				25.07	1.65	12.70	4.70	0.025

Supplementary Table 6. Bayesian estimates of intercept and slope from regressions of temperature (°C) versus time. Independent variables are: mean monthly temperature in February (yearly minimum), March (best predictor for barnacle recruitment), August (yearly maximum and best predator for mussel recruitment), the yearly median, yearly mean and yearly mean for the decade starting in 2003.

Model	Term	posterior mean	lower 95% credible limit	upper 95% credible limit	effective sample size	pMCMC
Minimum	Intercept	-126.372	-356.922	59.635	2000	0.208
	February	0.064	-0.029	0.178	2000	0.202
March	Intercept	-121.946	-356.817	98.653	2000	0.281
	March	0.062	-0.048	0.179	2000	0.268
Maximum	Intercept	-154.390	-280.116	-26.253	2000	0.023
	August	0.085	0.022	0.148	2000	0.016
Median	Intercept	-211.310	-331.819	-69.504	1964	0.004
	Median	0.110	0.039	0.170	1964	0.003
Mean	Intercept	-129.900	-249.000	-0.488	2000	0.044
	Mean	0.069	0.005	0.128	2000	0.034
Mean (2004 - 2014)	Intercept	-244.400	-492.200	27.210	2000	0.063
	Mean	0.126	-0.009	0.249	2000	0.060

Supplementary Table 7. Bayesian estimates of random effects from Poisson regressions shown in Figure 1. Models done using number per m^2 for LL, LO, TT and NL; square root of number per 36 cm^2 for SB; number per 40 cm^2 for ME. Codes are: LL=L. *littorea*, LO=*L. obtusata*, TT=*T. testudinalis*, NL=*N. lapillus*, SB=*S. balanoides*, ME=*M. edulis*.

Species	Term	posterior mean	lower 95% credible limit	upper 95% credible limit	Percentage of variation	Effective sample size
LL	Site	0.359	0.112	0.758	49.81%	1761
	units	0.362	0.298	0.432	50.19%	2000
LO	Site	0.462	0.116	0.947	30.46%	2000
	units	1.056	0.855	1.278	69.54%	2000
TT	Site	2.533	0.464	5.721	60.66%	2000
	units	1.643	1.018	2.313	39.34%	2000
NL	Site	1.072	0.275	2.221	37.92%	2000
	units	1.754	1.265	2.236	62.08%	1873
SB	Site	0.308	0.038	0.683	23.71%	2065
	units	0.990	0.671	1.303	76.29%	2000
ME	Site	0.661	0.177	1.441	37.51%	2000
	units	1.101	0.868	1.374	62.49%	2000

Supplementary Table 8. Correlations over time among abundances of gastropods, and recruitment of mussels and barnacles based on residuals from fitted models. Panels A and C are correlations, and panels B and D are P-values (upper triangle) and sample sizes (lower triangle). Codes are: LL=L. *littorea*, LO=*L. obtusata*, TT=*T. testudinalis*, NL=*N. lapillus*, SB=*S. balanoides*, ME=*M. edulis*. Correlations and P-values are shown in bold when $P < 0.05$; tests are two-sided.

A: Pearson's correlations

	LL	LO	TT	NL	SB	ME
LL	1.000	**0.137**	**0.300**	0.018	0.109	0.015
LO		1.000	0.074	0.313	0.012	**-0.169**
TT			1.000	0.055	-0.020	-0.005
NL				1.000	0.144	-0.081
SB					1.000	-0.033
ME						1.000

C: Spearman's correlations

	LL	LO	TT	NL	SB	ME
LL	1.000	0.013	**0.418**	**0.154**	**0.130**	0.028
LO		1.000	-0.050	**0.262**	-0.125	**-0.270**
TT			1.000	**0.369**	0.028	**0.333**
NL				1.000	0.092	0.008
SB					1.000	0.046
ME						1.000

B: P-values and sample sizes

	LL	LO	TT	NL	SB	ME
LL		**0.020**	**>0.001**	0.764	0.097	0.828
LO	288		0.252	**>0.001**	0.849	**0.016**
TT	239	239		0.394	0.783	0.948
NL	288	288	239		0.027	0.251
SB	235	235	196	235		0.649
ME	202	202	169	202	197	

D: P-values and sample sizes

	LL	LO	TT	NL	SB	ME
LL		0.830	**>0.001**	**0.009**	**0.047**	0.689
LO	288		0.438	**>0.001**	0.056	**>0.001**
TT	239	239		**>0.001**	0.695	**>0.001**
NL	288	288	239		0.161	0.906
SB	235	235	196	235		0.521
ME	202	202	169	202	197	
