# Supplementary material for: Declines over the last two decades of five intertidal invertebrate species in the western North Atlantic
Source: Commun Biol. 2020 Oct 20;3:591. doi: 10.1038/s42003-020-01326-0 (PMC7576203; doi:10.1038/s42003-020-01326-0)
Supplement: Supplementary file 3 — Description of Additional Supplementary Files [file 42003_2020_1326_MOESM3_ESM.pdf]

Description of Additional Supplementary Files

File Name: Supplementary Data 1
Description: Means for water temperature, pH, saturation ratio (Omega), snail abundances, and recruitment of barnacles and mussels. Columns labeled by month are average water temperatures at one m depth from the F01 buoy. Data for pH and omega are from published data[19]. Year column lists spring (e.g. 1997.3) and summer (e.g. 1997.5) sampling. Remaining columns are averages for abundances of *Littorina littorea* (LL), *Littorina obtusata* (LO), *Tectura testudinalis* (TT), *Nucella lapillus* (NL) and for recruitment of *Semibalanus balanoides* (SB) and *Mytilus edulis* (ME). Abundances are averages per 0.25 m2, which is the size of the sampling quadrat. Recruitment averages for *S. balanoides* are per surface area of sampling plate (39.6 sq. cm.) and for *M. edulis* are per sampling unit (approx. 40 cm^2). NA indicates missing data. Temperature data for April 2017, May 2017 and October 2018 were imputed
