# Supplementary material for: Declines over the last two decades of five intertidal invertebrate species in the western North Atlantic
Source: Commun Biol. 2020 Oct 20;3:591. doi: 10.1038/s42003-020-01326-0 (PMC7576203; doi:10.1038/s42003-020-01326-0)
Supplement: Supplementary file 5 — Reporting Summary [file 42003_2020_1326_MOESM5_ESM.pdf]

nature research

Corresponding author(s): Peter S Petraitis

Last updated by author(s): Jul 1, 2020

Reporting Summary

Nature Research wishes to improve the reproducibility of the work that we publish. This form provides structure for consistency and transparency in reporting. For further information on Nature Research policies, see our Editorial Policies and the Editorial Policy Checklist.

Statistics

For all statistical analyses, confirm that the following items are present in the figure legend, table legend, main text, or Methods section.

n/a	Confirmed	
☐	☒	The exact sample size (*n*) for each experimental group/condition, given as a discrete number and unit of measurement
☐	☒	A statement on whether measurements were taken from distinct samples or whether the same sample was measured repeatedly
☐	☒	The statistical test(s) used AND whether they are one- or two-sided<br>*Only common tests should be described solely by name; describe more complex techniques in the Methods section.*
☐	☒	A description of all covariates tested
☐	☒	A description of any assumptions or corrections, such as tests of normality and adjustment for multiple comparisons
☐	☒	A full description of the statistical parameters including central tendency (e.g. means) or other basic estimates (e.g. regression coefficient) AND variation (e.g. standard deviation) or associated estimates of uncertainty (e.g. confidence intervals)
☐	☒	For null hypothesis testing, the test statistic (e.g. *F*, *t*, *r*) with confidence intervals, effect sizes, degrees of freedom and *P* value noted<br>*Give P values as exact values whenever suitable.*
☐	☒	For Bayesian analysis, information on the choice of priors and Markov chain Monte Carlo settings
☒	☐	For hierarchical and complex designs, identification of the appropriate level for tests and full reporting of outcomes
☒	☐	Estimates of effect sizes (e.g. Cohen's *d*, Pearson's *r*), indicating how they were calculated

Our web collection on statistics for biologists contains articles on many of the points above.

Software and code

Policy information about availability of computer code

Data collection	No software was used
Data analysis	R script for analyses are available online at:https://doi.org/10.6073/pasta/7ad4e0c85ff585199cb2a007eb291c9d.

For manuscripts utilizing custom algorithms or software that are central to the research but not yet described in published literature, software must be made available to editors and reviewers. We strongly encourage code deposition in a community repository (e.g. GitHub). See the Nature Research guidelines for submitting code & software for further information.

Data

Policy information about availability of data

All manuscripts must include a data availability statement. This statement should provide the following information, where applicable:

- Accession codes, unique identifiers, or web links for publicly available datasets
- A list of figures that have associated raw data
- A description of any restrictions on data availability

The data sets generated and analyzed during the current study are available online at: https://doi.org/10.6073/pasta/7ad4e0c85ff585199cb2a007eb291c9d.

Field-specific reporting

Ecological, evolutionary & environmental sciences study design

All studies must disclose on these points even when the disclosure is negative.

Study description	The data used in this paper are the control plots from a larger long-term study. The description of the treatment levels used in the larger study are not described here. There are 12 sites and at each site there is a single control plot. Sites are nested within four bays on Swans Island, Maine, with three sites in each bay. Sites within bays were at least 750 m apart. Bay was ignored as a factor in the analyses. The plots were established between 22 June and 17 August 1996 . Center of plots were marked with two stainless steel reference bolts which were anchored in drilled holes. Plots were left unmanipulated thereafter.
Research sample	Abundances of gastropods (Testudinalia testudinalis, Littorina littorea, Littorina obtusata and Nucella lapillus) were counted using three or five 50 × 50 cm quadrats per plot. Quadrats were haphazardly placed within 0.25 m of the reference bolts. Data used in the analyses were means of quadrats per plot per sampling.<br><br>Plates used for barnacle recruitment were resin castings made from latex molds of natural granite rock (Fig. 1). Plates were disk-shaped; approximately 0.67 cm thick and 7.1 cm in diameter (39.6 cm2 in area). Barnacle recruitment in Maine begins in March and continues through May and so plates for barnacle recruitment were usually placed into the plots in late March (average date: 26 March; range: 1 March – 31 May) and usually collected in late May (average date: 22 May; range: 3 April – 28 June) for an average of 57 days (range: 27–77 days). A complete count of cyprid larvae and newly metamorphosed spat on each plate was done within a day of collection using a dissecting scope at 25×. Data file provides total count per plate.<br><br>Settling mussel larvae tend to attach initially to filamentous algae and so fibrous pads were used as substrates for recruitment. Pads were approximately 5 × 5 cm and cut from furnace filters (range of fiber diameter = 0.3–0.5 mm). Pads were wrapped in a 5 cm × 13 cm piece of plastic mesh (width of mesh opening: 4.6–5.0 mm; diameter 1.5 mm), which was closed with two cable ties. On Swan's Island, mussels begin to recruit in June, peak in July and early August, and continue through. Pads were usually placed out in late May (average date: 27 May; range: 1 May – 27 June) and usually collected in late August (average date: 21 August; range: 8 August – 30 August) for an average of 85 days (range: 67–108 days). Upon collection, pads were preserved in 70% isopropyl alcohol. For counting, pads were torn open and rinsed in water over 425 µm and 300 µm sieves. In most years, mussels trapped on each sieve were rinsed onto a tray with a 2 × 2 cm grid of 32 squares (4 × 8), and mussels were counted in five (1998, 2000, and 2001) or ten (2003–2007) randomly chosen squares. In 1997, counts were collected as 10 circular fields, 1 cm in diameter (Dudgeon and Petraitis 2001); raw totals were multiplied by 5.092 to give numbers per 40 cm2. In 1999, mussels in the entire tray were counted. Counts in data file are reported as numbers per 40 cm2 and rounded to the nearest whole number (i.e., equivalent to the total of ten 2 × 2 cm squares).<br><br>Environmental data for pH, aragonite saturation ratio (ΩAR) and temperature were extracted from published papers and online databases. Yearly averages in pH and ΩAR from 1997 to 2014 were taken from published figures.. Monthly mean temperatures at 1 m depth and from 2002 to 2018 were downloaded from NERACOOS for the three buoys that are the closest to Swans Island (http://neracoos.org/datatools/climatologies_display). The buoys are E01 (Central Maine Shelf), F01 (Penobscot Bay) and I01 (Eastern Maine Shelf). Averages among the buoys were highly correlated and all three pairwise correlations were greater than 0.942. Buoy F01 was missing the fewest observations (April 2007, May 2007 and October 2008) and so these data were used for analysis.
Sampling strategy	Abundances of gastropods are typically sampled by marine ecologists using a 50 x 50 cm quadrat frame. We used the mean of 3-5 quadrats per plot per sampling as a datum; this provided an accurate estimate of abundance. We switched from doing 5 quadrats per plot to three quadrats per plot in the early 2000s because of time constraints of sampling at low tide. We found no loss of precision. We have found use of mean counts for gastropods and totals from recruitment surfaces for barnacles and mussels gives us sufficient power to detect differences among years and among treatment levels. Collection of environmental data was constrained by what is available online.
Data collection	See Research sample section for decription of data collection. Data has been primarily collected by the authors (Petraitis and Dudgeon) and our field assistant (Erika Rhile). Others have helped at times, but at least one of the three of us have always been present. Collection of data in the field can be dangerous because of slippery conditions and at least two people were present during data collection.
Timing and spatial scale	Collection of data on abundances of gastropods and recruitment of barnacles and mussels started in 1997 and has continued annually since then. This paper includes data through 2018. Data were not collected in 2009. Gastropod abundances were sampled once a year in the summer. In 1997, 1998, and 2000, abundances were also sampled in the spring. Data for mussel recruitment are missing for 1998, 2000, 20002 and 2010. Data for barnacle recruitment are missing for 2000 and 2010. Data for monthly mean temperatures were available from 2002 to 2018; data for April 2007, May 2007 and October 2008 were missing. Yearly means for pH and aragonite saturation ratio were available from 1997 to 2014.<br><br>The 12 sites are nested within four bays; three sites in each bay. Two bays are on the north side of Swans Island, Maine and two are on the south side. Straight line distances among the bays range from 2.5 km to 5.0 km although the distance by water greatly exceeds those distances. Sites within bays are at least 750 m apart.
Data exclusions	In three cases, data were transformed or dropped because models using the full dataset failed to converge. Data for barnacle (S. balanoides) recruitment were square-root transformed. For mussel recruitment (M. edulis), 1997 data were dropped because of extremely large values (average 1161 per 40 cm2, range: 32-5424). For limpet abundance (T. testudinalis), data from two sites were dropped because limpets were absent or very rare from 2000 to 2018, and the datum for one site in 1998 for which abundance was >10 per m2.
Reproducibility	Because data are from a long-term study, we have not repeated the study. Our estimates for abundances of gastropods are well within the ranges reported at other locations throughout the north Atlantic.

Randomization	As mentioned in the Study Description, the data are from control plots that are part of a larger experimental study. For this larger study, started in 1996, sites were chosen to be as similar as possible in terms of slope aspect and species composition, and large enough to accommodate five levels of treatment (controls plus four manipulations). Treatment levels within sites were at least 20 m apart. Sites were also chosen based on accessibility. As mentioned before, sites were nested within bays.
Blinding	Blinding was not done because we had no expectation that there would be long-term declines. We only noticed the patterns when we began to work up the data for the entire study. We opted to use only the control plots for this paper as to not confound the pattern with our experimental manipulations.

Did the study involve field work? [x] Yes [ ] No

Field work, collection and transport

Field conditions	Data were collected on low tides regardless of the weather conditions, which included days of rain, fog and high winds. Data were not collected if there was a threat of lightening. Spring and summer temperatures range form the high 40s to low 70s. F.
Location	Swans Island, Maine, USA is within a box defined by: N: 44.191442; S: 44.12869; E: -68.38357; W: -68.503708. Plots are in the mid-intertidal zones of sheltered bays and between 0.3 and 1.0 m above mean low water. The mid-intertidal shores are protected from wave surge and dominated by the rockweed Ascophyllum nodosum. The shoreline at the different sites are a mixture of granite and basalt outcrops, boulder fields, and some muddy patches in the most protected sites. GPS positions of all plots, including the control plots used here can be found in Supplement 1 in Petraitis and Dudgeon (2015, Ecology 96:3186-3196).
Access & import/export	Gastropod abundances were counted in place; animals were not removed. Recruits of barnacles and mussels were collected on surfaces that were removed from the field to be count. Once counted, surfaces were cleaned and recruits removed. No permits were required. We would note that a single cleaning of a boat bottom to remove mussels, barnacles and other fouling organisms kills orders of magnitude more far individuals than our sampling
Disturbance	The only disturbance was trampling of seaweeds and other organisms as we walked across the intertidal shore to reach our plots.

Reporting for specific materials, systems and methods

We require information from authors about some types of materials, experimental systems and methods used in many studies. Here, indicate whether each material, system or method listed is relevant to your study. If you are not sure if a list item applies to your research, read the appropriate section before selecting a response.

Materials & experimental systems

n/a	Involved in the study	
[x]	[ ]	Antibodies
[x]	[ ]	Eukaryotic cell lines
[x]	[ ]	Palaeontology and archaeology
[ ]	[x]	Animals and other organisms
[x]	[ ]	Human research participants
[x]	[ ]	Clinical data
[x]	[ ]	Dual use research of concern

Methods

n/a	Involved in the study	
[x]	[ ]	ChIP-seq
[x]	[ ]	Flow cytometry
[x]	[ ]	MRI-based neuroimaging

Animals and other organisms

Policy information about studies involving animals; ARRIVE guidelines recommended for reporting animal research

Laboratory animals	No lab animals were used.
Wild animals	No wild animals were collected
Field-collected samples	Counts of mussel and barnacle recruits were done in the lab. See Research Sample section for details on how recruits were collected and counted.
Ethics oversight	No ethical oversight is required for counting gastropods in the field and for sampling recruits of mussels and barnacles.

Note that full information on the approval of the study protocol must also be provided in the manuscript.
